# Distinct Dynamics of Migratory Response to PD-1 and CTLA-4 Blockade Reveals New Mechanistic Insights for Potential T-Cell Reinvigoration following Immune Checkpoint Blockade

**DOI:** 10.3390/cells11223534

**Published:** 2022-11-08

**Authors:** Fateme Safaeifard, Bahram Goliaei, Amir R. Aref, Mohammad-Hadi Foroughmand-Araabi, Sama Goliaei, Jochen Lorch, Russell W. Jenkins, David A. Barbie, Seyed Peyman Shariatpanahi, Curzio Rüegg

**Affiliations:** 1 Laboratory of Biophysics and Molecular Biology, Institute of Biochemistry and Biophysics (IBB), University of Tehran, Tehran 1417614411, Iran; 2 Department of Medical Oncology, Dana-Farber Cancer Institute, Boston, MA 02215, USA; 3 Xsphera Biosciences Inc., Boston, MA 02210, USA; 4 Department of Mathematical Sciences, Sharif University of Technology, Tehran P.O. Box 11155-9415, Iran; 5 Faculty of New Sciences & Technologies, University of Tehran, Tehran 1439957131, Iran; 6 Robert H. Lurie Comprehensive Cancer Center, Northwestern University, Chicago, IL 60611, USA; 7 MassGeneral Cancer Center, Massachusetts General Hospital, Harvard Medical School, Boston, MA 02114, USA; 8 Belfer Center for Applied Cancer Science, Dana-Farber Cancer Institute, Boston, MA 02115, USA; 9 Laboratory of Experimental and Translational Oncology, Department of Oncology, Microbiology, and Immunology, Faculty of Sciences and Medicine, University of Fribourg, 1700 Fribourg, Switzerland

**Keywords:** organotypic tumor culture, immune checkpoint blockade, T-cell migration, heterogeneous random walks, tumor–immune interaction, delayed dynamics

## Abstract

Cytotoxic T-lymphocyte-associated antigen 4 (CTLA-4) and programmed cell death protein 1 (PD-1), two clinically relevant targets for the immunotherapy of cancer, are negative regulators of T-cell activation and migration. Optimizing the therapeutic response to CTLA-4 and PD-1 blockade calls for a more comprehensive insight into the coordinated function of these immune regulators. Mathematical modeling can be used to elucidate nonlinear tumor–immune interactions and highlight the underlying mechanisms to tackle the problem. Here, we investigated and statistically characterized the dynamics of T-cell migration as a measure of the functional response to these pathways. We used a previously developed three-dimensional organotypic culture of patient-derived tumor spheroids treated with anti-CTLA-4 and anti-PD-1 antibodies for this purpose. Experiment-based dynamical modeling revealed the delayed kinetics of PD-1 activation, which originates from the distinct characteristics of PD-1 and CTLA-4 regulation, and followed through with the modification of their contributions to immune modulation. The simulation results show good agreement with the tumor cell reduction and active immune cell count in each experiment. Our findings demonstrate that while PD-1 activation provokes a more exhaustive intracellular cascade within a mature tumor environment, the time-delayed kinetics of PD-1 activation outweighs its preeminence at the individual cell level and consequently confers a functional dominance to the CTLA-4 checkpoint. The proposed model explains the distinct immunostimulatory pattern of PD-1 and CTLA-4 blockade based on mechanisms involved in the regulation of their expression and may be useful for planning effective treatment schemes targeting PD-1 and CTLA-4 functions.

## 1. Introduction

Immune-regulatory mechanisms modulate the immune response, primarily to allow immune recovery and elude autoimmune reactions. Several regulatory mechanisms involved in physiological immune tolerance are exploited by malignant tumors to escape immune rejection. Among them, immunosuppressive signaling pathways have a critical role in the regulation of chronic immune responses. Cytotoxic T-lymphocyte–associated antigen 4 (CTLA- 4) and programmed death 1 (PD-1), two negative co-stimulatory receptors, attenuate T-cell activation mainly through intrinsic cellular mechanisms [1].

The CTLA-4 molecule effectively blocks the CD28-dependent co-stimulatory pathway through the competitive inhibition of CD80 and CD86 ligands thanks to its higher affinity for CD28. CD28-dependent signals are mainly mediated by phosphatidyl inositol 3-kinase (PI3K) and Grb2 signaling molecules, which have established contributions to actin-based cell movement alongside other effector functions [2,3,4,5,6,7,8]. In addition, ligand-bound CTLA-4 releases an inhibitory signal that interrupts CD28 and T-cell receptor (TCR) signaling cascades [1,3].

PD-1 engagement by its specific ligands (PD-L1 and PD-L2) directly suppresses the TCR signaling cascade through the dephosphorylation and deactivation of its coupled components following the recruitment of specific protein-tyrosine phosphatases. However, most of the affected molecules are also involved in the regulation of cell migration, actin polymerization, and T-cell anergy [8,9,10,11]. In addition, evidence suggests that PD-1 and CTLA-4 converge on the modulation of the CD28 signaling pathway [12]. 

The blockade of PD-1 and CTLA-4 receptors attenuates their downstream signals, which enables T cells’ reinvigoration, boosting the anti-tumor response. The clinical success of checkpoint-inhibition-based cancer treatments is beholden to the fundamental research that provided mechanistic views on immune regulation and tolerance. In spite of some undeniable successes, checkpoint inhibitors are only effective in selected cancer types (e.g., lung, melanoma, and bladder), with variable performance for patients [13]. Thus, improving the therapeutic efficacy of PD-1 and CTLA-4 blockade would benefit from more detailed mechanistic insights, particularly on the comparative aspects of checkpoint functions that intertwined with the coordinated nature of immunoregulatory mechanisms and their mutual compensatory relationships [1,14,15,16]. 

Despite the overlapping activities of the PD-1 and CTLA-4 receptors and downstream pathways, many studies indicate that each pathway has some peculiar specificities, for example, the timing of their action, their corresponding immune cell populations, their predominant operating environments, and downstream signal-transducing molecules [17,18,19,20]. While TCR activation upregulates both receptors’ expression, the existence of CTLA-4 cytoplasmic reserves provides this receptor with the exclusive possibility of rapid intracellular trafficking and translocation to the cell surface to adjust its membrane level according to the TCR signal strength [21,22]. These molecular and mechanistic characteristics can eventually manifest themselves in the dynamics of tumor–T-cell interaction. However, in spite of its potential clinical relevance, comparative studies characterizing the kinetics of checkpoint-induced immune inactivation remained rare. 

Mathematical modeling represents a valuable approach for identifying mechanisms underlying complex biological systems and proposing new hypotheses. The complexity of a system is indicated by the complex endogenous patterns of the system itself and originates from the nonlinear characteristics and temporal delays of its interacting components [23].

Several previous studies have considered various components involved in the dynamics of tumor–immune-cell interaction in the presence of immunostimulatory drugs or checkpoint inhibitors for the model-based prediction of key parameters and the development of optimal therapeutic strategies [24,25,26,27,28,29,30,31,32,33]. For example, a mathematical framework highlighted the synergistic effect of combined radiotherapy and checkpoint blockade based on the immunogenicity of irradiation and the intensification of abscopal effects by the concurrent checkpoint blockade [24]. A prognostic model describing the long-term response (tumor burden) to checkpoint inhibitors was developed and calibrated with a large-scale literature-derived patient cohort. The model parameters provided a sensitivity threshold for the evaluation of responders and revealed biomarkers for predicting treatment efficiency [28,29]. Some mathematical models integrated pharmacological kinetic information to predict effective drug doses in addition to optimal treatment scenarios [25,30]. Particular attention has been dedicated to investigating the interesting dynamical phenomena in tumor–immune interaction and its implications in the immunotherapy of cancer [31,32,33].

While most of the models consider cell–cell interaction networks, the integration of the related molecular processes may resolve the relative contributions of intracellular peculiarities to the modulation of the response to the inhibition of checkpoint pathways.

Furthermore, extensive clinical efforts to identify biomarkers of the response to PD-1 and CTLA-4 blockade are hampered by complex patterns that are dependent on pretreatment conditions and the scheduling of treatments [13,34]. Molecular findings demonstrated that PD-1 signaling employs a more exhaustive molecular cascade to disturb PI3K/AKT signaling and displays a more efficient cell-level function compared with the CTLA-4 pathway [19]. Nevertheless, clinical observations indicate broader immune restoration and more significant TCR repository enrichment following CTLA-4 inhibition. Consistently, the prevalence of immune-related adverse effects is one of the major challenges in the anti-CTLA-4-based immunotherapy of cancer [35,36,37,38]. Contrary to expectation, this relatively broad immune restoration does not always lead to a superior therapeutic response and tumor shrinkage compared with PD-1 blockade [37,39]. These controversial patterns of responses to checkpoint blockade make the identification of robust clinical biomarkers more challenging.

To mathematically resolve these controversies in clinical outcomes, here, we explore the kinetic pattern governing PD-1 and CTLA-4 functions to address the possible therapeutic limitations originating from the coordinated cell-level response to these pathways.

For this purpose, we used a previously developed ex vivo system that recapitulates the tumor microenvironment, allowing the precise monitoring of the tumor response to immune checkpoint blockade [40,41]. The system consists of a 3D microfluidic culture of organotypic tumor spheroids derived from patient samples that retain autologous immune cells. The applied microfluidic device allowed for controlled treatment with anti-PD-1 and anti-CTLA-4 antibodies, as well as a short-term evaluation of the response to these inhibitors. Previously published data based upon cytokine measurements and immune profiling validated the system as a novel platform for biomarker identification and the systematic evaluation of checkpoint blockade outcomes [42]. 

Here, using time-lapse imaging and single-cell tracking, we obtained time series of immune cell movements in three conditions of individual and combined PD-1 and CTLA-4 blockade to derive the state of immune cell activity and follow their temporal response within an immunosuppressive tumor environment. The retrogressive movement of immune cells, as well as the distinct dynamics of their response to PD-1 and CTLA-4 pathway modulation, was delicately captured by extracting temporal variations in parameters in a heterogeneous random walk model. Finally, a model of tumor–immune interaction based on a system of ordinary differential equations revealed how the characteristic dynamics of PD-1 and CTLA-4 activation potentially imposes limitations on the tumor response to PD-1 blockade. 

## 2. Materials and Methods

**Figure 1:**
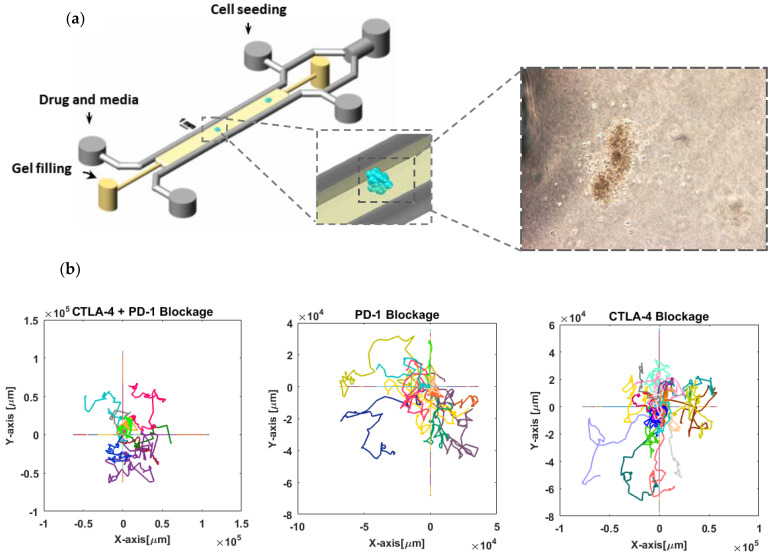
**Tracking lymphocyte migration in 3D organotypic tumor culture.** (**a**) Schematic view of the microfluidic device used for 3D culture of thyroid tumor spheroids (scale bar shows 1 mm). Patient-derived tumor spheroids containing autologous immune cells were loaded in a collagen medium in the central channel of the device and subjected to anti-PD-1 or anti-CTLA-4 antibody treatment. A representative bright-field image of a thyroid tumor spheroid shows cells migrated into the surrounding collagen. (**b**) Cell trajectories in three different conditions of pathway inhibition. A total of 12, 28, and 9 migrating cells were tracked in the conditions of anti-PD-1, anti-CTLA-4, and combination exposure, as indicated. For each experiment, individual trajectories are shown with distinct colors, and all trajectories started from the same point (coordinate center) that is overlaid on the same graph. Time-lapse images were captured every 15 min for at least 130 h.

**Cell migration tracking:** Patient-derived organotypic tumor spheroids were obtained as previously reported [42] and studied for the migration activity of immune cells in response to checkpoint blockade (Figure 1a). Three and ten spheroid cultures derived from melanoma patient biopsies responsive to PD-1/PD-L-1 blockade were investigated for anti-CTLA-4 (Nivolumab) + anti-PD-1 (Ipilimumab) treatment or the exclusive inhibition of PD-1, respectively. The response to CTLA-4 inhibition was analyzed in five thyroid tumor cultures derived from a patient nonresponsive to PD-1/PD-L-1 blockade. The same sample also accounted for six control experiments with no drug treatment. The detectable moving cells were tracked in each experiment (Appendix A) to extract cell trajectories and related parameters, such as velocity and persistence, using the Tracking package of IMARIS software.

**Calculation of mean-square displacement of the cells:** Mean-square displacement (MSD) was evaluated as a substantial criterion to investigate the diffusive behavior of the cells. The MSD of a walker is defined as follows:

(1) $$R^{2}\left(\tau \right)=\underset{t\to \infty }{lim}\langle {\left(X\left(t+\tau \right)-X\left(t\right)\right)}^{2}\rangle ;$$

where |X(t+τ)−X(t)| corresponds to the displacement of the walker between two consecutive steps. The parameters τ and t refer to the time interval and total time of the movement, respectively. The MSDs were calculated for the cell trajectories as well as simulated ones to check their correspondence and evaluate the model of migration.

**Figure A2:**
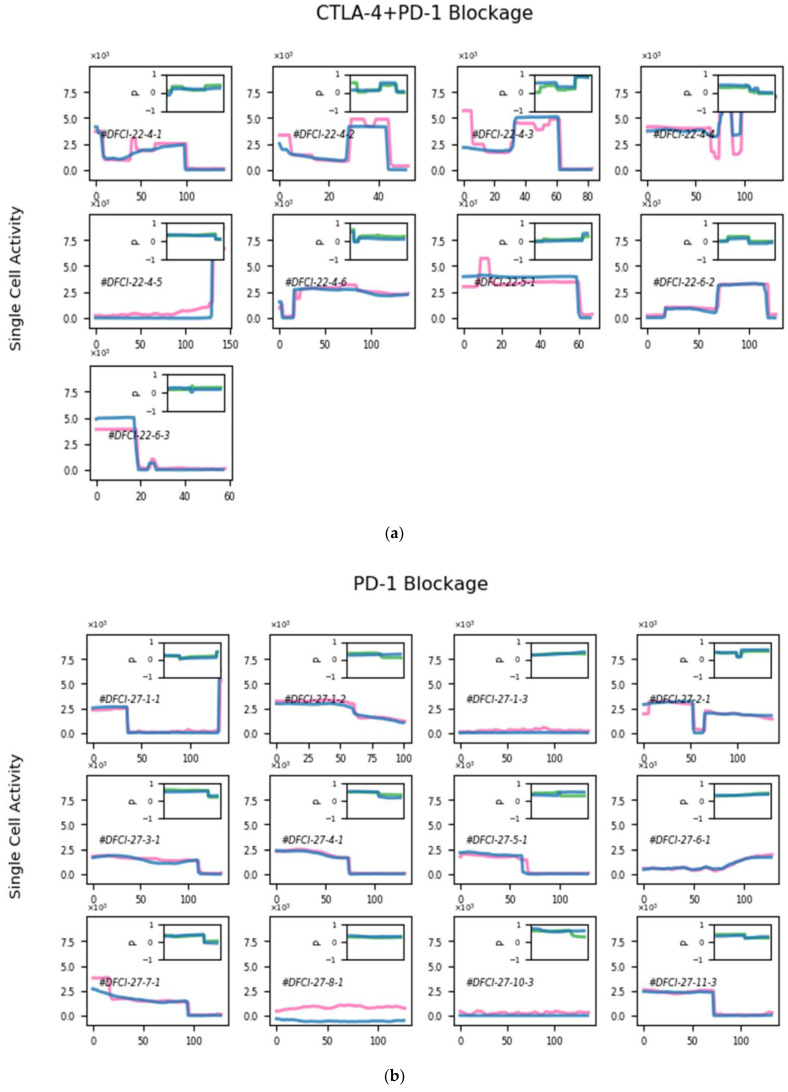

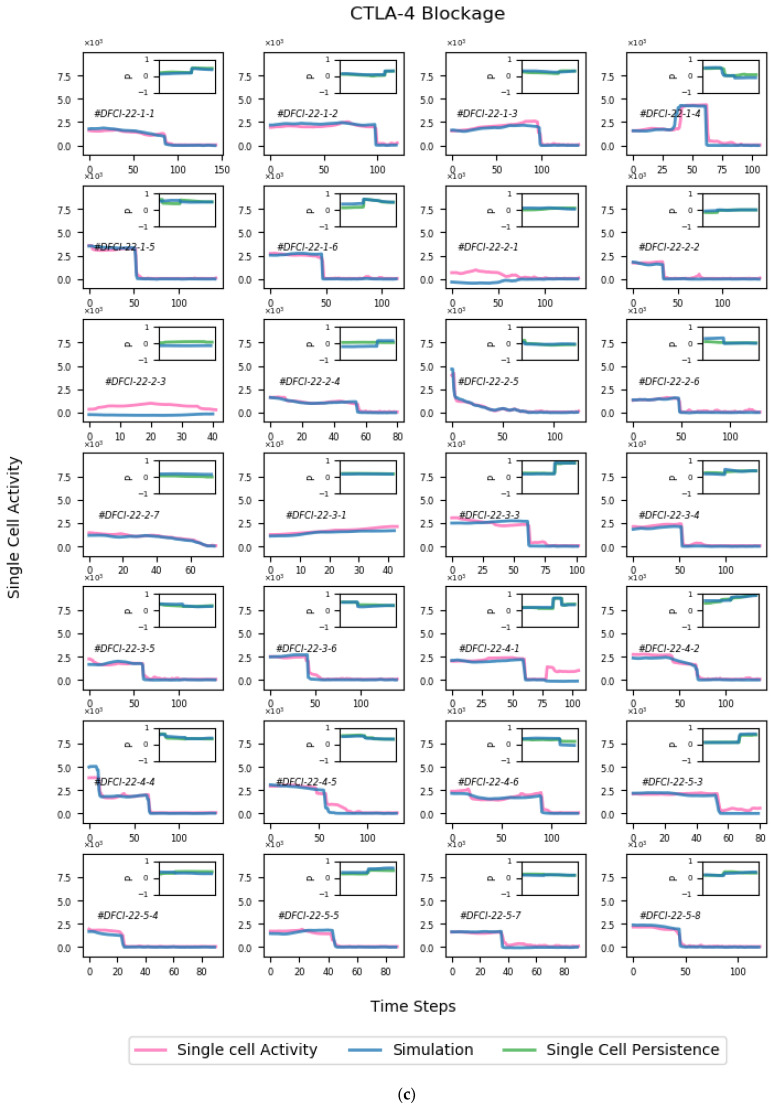
**Single-cell parameters of AR-1 model of migration in combined inhibition of PD-1 and CTLA-4 pathways (a) as well as PD-1 (b) and CTLA-4 blockade (c).** The Bayesian inference method was applied to time series of cell trajectories, and the mean values of the inferred activity parameter are shown for each cell (pink curves). The results of parameter estimation for AR1-simulated trajectories (blue curves) show good agreement with those derived from the experiment. Insets: Single-cell persistence of the cells (dark green curves) and simulation-derived ones (light green curves). The # notes indicate IDs of patients from Dana–Farber Cancer Institute, the number of tumor spheroids, and the tracked cell number, in that order.

**Bayesian inference method for parameter estimation of the migration model:** We used heterogeneous random walks to model the migration of tumor-infiltrating (antigen-experienced) lymphocytes and the detection of temporal variations in model parameters affected by immune checkpoint activation. We applied an algorithm designed and implemented by Metzner, C. et al. [43] for the sequential estimation of the random walk model parameters based on the Bayesian inference method. Accordingly, the displacements are calculated using cell velocities in each time step, and the time-varying displacement vector is described according to a first-order autoregressive model:

(2) $$u\left(t\right)=q\left(t\right)u\left(t-1\right)+a\left(t\right)n_{\left(0;1\right)}\left(t\right);$$

where the parameter q(t) represents the time-varying persistence of the cells and ranges between −1 and 1, from anti-persistent to persistent random motion. The parameter a(t) corresponds to the motion activity, namely, the intensity of noise in the random walk process. The random noise vector $n=\left(n_{x};n_{y}\right)$ is taken from an uncorrelated Gaussian distribution with unit variance. The likelihood function of the stochastic process conducts the sequential Bayesian method to infer the joint distribution of the parameters for each time step. Single-cell activity and persistence are given in Figure A2. See Appendix C for more details.

**Simulation of cell trajectories:** The time-varying autoregressive model of the first order was evaluated for the analysis of cell movements. In this way, we used the estimated activity and persistence of the cells to simulate trajectories based on the same autoregressive process. The model parameters were further estimated for the simulated trajectories to be compared with the input parameters and to confirm the accuracy of the applied algorithm. The subsequent statistical verification of the simulated trajectories determines the accuracy of this modeling paradigm.

**Mathematical model hypothesis**: A set of biological assumptions was considered to infer a mathematical model of checkpoint-induced immune modulation. These assumptions outline the theoretical underpinning of the work and reduce the parameters and equations to those sufficient for a rational description of the experimental data. 

*Accessibility of tumor cells*: The experimental results indicate that even in the case of the two-pathway blockade, a fraction of tumor cells would survive. We supposed that this is because of the limited accessibility of tumor-infiltrating lymphocytes to the population of tumor cells, a well-known phenomenon that plays a role in tumor-associated immune resistance [44]. Assuming a spherical geometry for tumor spheroids, Equation (7) relates the accessible fraction of tumor cells to their total population [45].

*Temporal delay in surface expression of PD-1 receptor:* Most of the cellular CTLA-4 receptors are stored in cytoplasmic vesicles. The surface expression of CTLA-4 is quickly upregulated by membrane trafficking control and the translocation of this intracellular pool to the cell surface. In contrast, genetic and epigenetic mechanisms are mainly engaged in the regulation of PD-1 expression, which leads to a time delay in T-cell inactivation induced by the PD-1 pathway [21,22,46]. 

*Direct and indirect tumor*–*T*-*cell interaction:* The subpopulation of accessible tumor cells may be directly killed by T-lymphocytes and, in turn, stimulate PD-1 and CTLA-4 pathways via direct contact. *The* effects of T-cell exhaustion factors other than PD-1 and CTLA-4 (free radicals, secreted cytokines, oxygen limitation, etc.) are assumed to be dependent on the total population of tumor cells (regardless of the direct access to T cells) [47].

*Upregulation of PD-1 expression*: We assume that the sustained presence of cancer cell antigens in the cultured tumor microenvironment leads to the upregulation of PD-1 surface expression. The rate of this induction is considered to be constant (independent of the population size of tumor cells).

*No proliferation and recruitment in the 3D channel microenvironment:* No immune cell recruitment occurs in this experimental setup, and tumor cell proliferation can be ignored because of limited nutrient availability in this condition. 

*Lymphocyte activity modeling:* It is assumed that tumor cell death occurs in proportion to lymphocyte activity, which appears in their migratory behavior. Therefore, the dynamics of activity for tumor-interacting lymphocytes is modeled, instead of their population changes. 

*Marker of reinvigorated cells:* The reinvigoration of T cells is detectable based on their measured mobility behavior.

**Figure 2:**
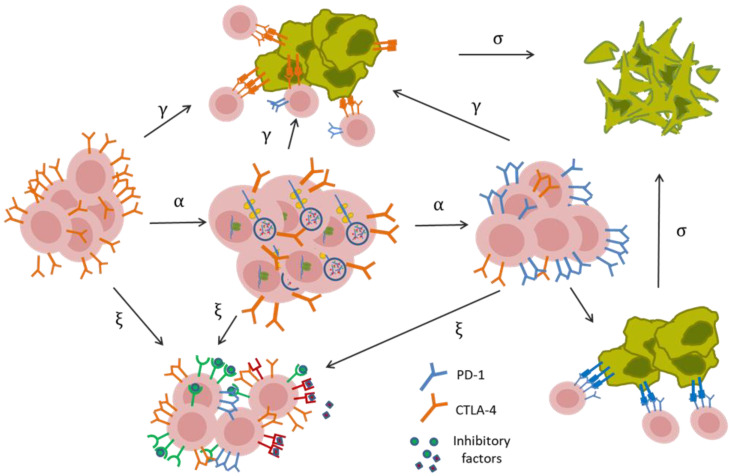
**Illustration of the relationships between the components of the mathematical model**. PD-1 and CTLA-4 receptors and other factors suppress lymphocyte activity in the tumor microenvironment. As a result of the cytoplasmic storage of preformed CTLA-4 molecules, the total population of active lymphocytes can express the CTLA-4 receptor with no time delay and can be potentially inactivated by this receptor at a gamma rate. Other inhibitory factors can also suppress lymphocytes, independent of PD-1 receptor signaling. The regulation of PD-1 expression occurs mainly at the genetic and epigenetic levels, resulting in a time delay in immune suppression triggered by PD-1 receptors. Concurrently, the cytotoxic activity of lymphocytes results in the death of tumor cells.

**Dynamical modeling of tumor-infiltrating lymphocyte inactivation induced by CTLA-4 and PD-1 signaling pathways:** As stated above, genetic, transcriptional, and translational regulation of PD-1 expression causes a few hours’ delay in lymphocyte inactivation triggered by this receptor. Considering the above assumption, along with other model hypotheses, and taking into account PD-1, CTLA-4, and other immunosuppressive factors in the tumor microenvironment, the dynamical model of tumor–T-cell interaction is schematically represented in Figure 2. 

According to the PD-1 expression state, the dynamical model comprises activities for three subpopulations of lymphocytes: lymphocytes with (1) unexpressed ($L_{u}$), (2) partially expressed ($L_{p}$), and (3) fully expressed $\left(L_{e}\right)$ PD-1 receptors. The subpopulation of lymphocytes lacking PD-1 receptors is converted to PD-1-expressing cells with a delay of the second order and the expression rate α. The conversion is then followed by PD-1-induced inactivation at a β rate in contact with the accessible tumor cells (Equations (3)–(6)).

It is assumed that all three subpopulations of active lymphocytes may be deactivated by CTLA-4 receptors at a γ rate, along with inhibitory factors other than PD-1 and CTLA-4 (i.e., radical formation and hypoxia) with a ξ rate of performance. The PD-1 signaling pathway can exclusively inhibit cells in the full expression state of PD-1 $\left(L_{e}\right)$. The cytotoxic activity of lymphocytes’ total population causes the death of tumor cells at a rate of σ (Equation (6)). 

Finally, the presumption of cell-to-cell contacts in CTLA-4 and PD-1 pathway activation, as well as the tumor cell death process, leads to a system of ordinary differential equations as follows:

(3) $$\frac{dL_{u}}{dt}=-\gamma C_{acc}L_{u}-\alpha L_{u}-\xi CL_{u},$$

(4) $$\frac{dL_{p}}{dt}=-\gamma C_{acc}L_{p}-\alpha L_{p}+\alpha L_{u}-\xi CL_{p},$$

(5) $$\frac{dL_{e}}{dt}=-\gamma C_{acc}L_{e}-\beta C_{acc}L_{e}+\alpha L_{p}-\xi CL_{e},$$

(6) $$\frac{dC}{dt}=-\sigma C_{acc}\left(L_{u}+L_{p}+L_{e}\right),$$

where the accessible proportion of tumor cells ($C_{acc}$) is calculated from the total population (C) as follows:

(7) $$C_{acc}=\frac{C}{1+\frac{C^{1/3}}{6}},$$

This equation is derived by assuming a simple spherical geometry for the tumor spheroids and the subsequent calculation of cells that hold a surface volume [45].

## 3. Results

### 3.1. Stochastic Modeling of T-Cell Migration

***T-cell trajectories***: The instances of T-cell trajectories under the condition of PD-1 and CTLA-4 blockade alone or in combination are illustrated in Figure 1b. The tracks represent 2D projections of cell trajectories in 3D cell cultures. These results show temporal variations in the T-cell step length, and for most of the cells, these variations display a decreasing trend in the time series of cell movements. No significant cell movement was observed in cultures without drug treatment (Appendix A).

**Figure 3:**
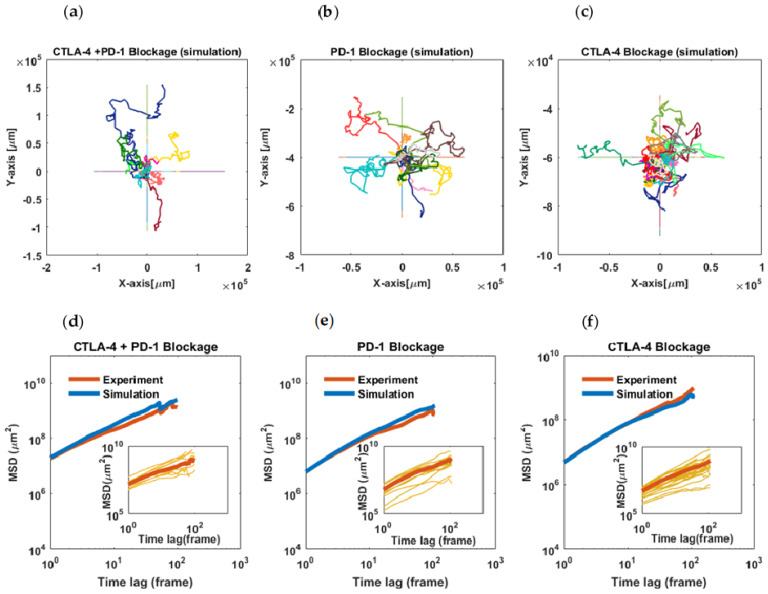
**Statistical analysis of cell migration and AR-1 model evaluation**. (**a**–**c**) Simulated trajectories using the AR-1 model with time-dependent parameters (activity and persistence) based on single-cell parameter changes estimated from experimental data. (**d**–**f**) Mean-square displacements (population-averaged) of tumor-infiltrating lymphocytes treated with anti-PD-1 or anti-CTLA-4 antibodies or a combination thereof (red curves). The MSDs show sub-diffusive behavior with an approximate power-law exponent of 0.83 for PD-1, 0.93 for CTLA-4, and 0.98 for combined inhibition. Blue curves represent the MSDs of simulated trajectories for each experiment. The intercepts of the curves indicate the highest value of the cell diffusion coefficient in the combined treatment (D = 7.3). The AR-1 model with changing parameters effectively reproduces the experimental MSDs obtained in each condition. Insets: MSD curves for individual cells. The last steps of the migration trajectories were removed because of error amplification in MSD calculations.

***Mean-square displacement of the cells:*** It was observed that the slope of the $MSD_{log-log}$ plots of the cells takes values smaller than one at many time intervals, which is a characteristic of sub-diffusive motions. Furthermore, in all three experimental conditions, the slopes of the curves have a decreasing trend and almost tend to zero (Figure 3d–f, blue curves).

***Temporal variation in cell migration parameters in checkpoint blockade experiments:*** We supposed that the observed migratory behavior can be explained by a model of random walks with time-dependent parameters. Based on this assumption, we used a first-order autoregressive model with time-varying parameters to model the studied cell movements. The sequential Bayesian inference method was applied to the cell trajectories to deduce the statistical parameters over the single-cell migration periods (Appendix B). 

For the evaluation of the autoregressive (AR-1) model of migration, the estimated parameters (time-varying persistence and activity of the cells) were used in an inverse manner to see if the model reproduces trajectories that statistically match the migration data (Figure 3a–c). In this way, the Bayesian method and applied algorithm were primarily assessed for the simulation of trajectories able to conserve the input parameters (Figure A2). Here, the MSDs of the simulated trajectories would show if the AR-1 model with changing parameters captures the migratory behavior of the cells. As shown in Figure 3d–f, there is good agreement between the population-averaged MSDs of the simulated time series and experimentally derived ones. 

**Figure 4:**
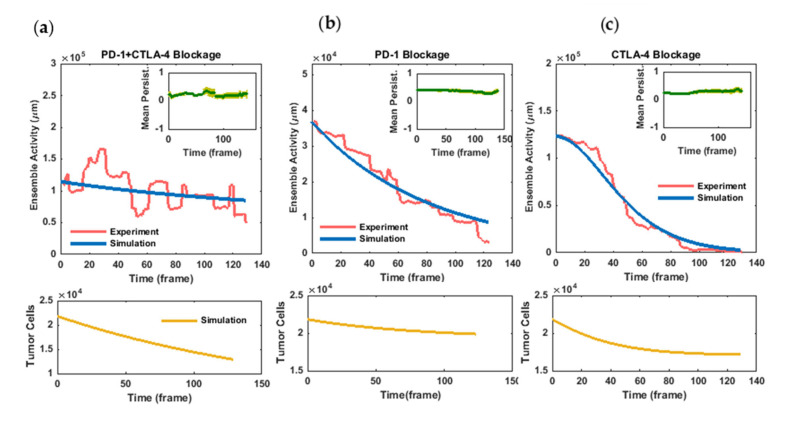
**Temporal variation in immune cell activity and dynamical model simulation in (a) combined as well as individual blockade of (b) PD-1 and (c) CTLA-4 pathways**. Immune cell activity displays a much lower initial value in PD-1 blockade compared with the two other conditions. Contrary to PD-1 blockade, the decrease in cellular activity in CTLA-4 blockade entails a delay of several hours, while in the combined inhibition of the pathways, the cells remain active up to the last frames of the assays. Dynamical model parameters were inferred by fitting the model (blue curves) to immune activity changes derived from cell migration data (pink curves). Tumor–immune profiling of the spheroids and live/dead cell staining confer an initial value of $T_{t_{0}}≅22000\pm 30\%$ and final values of $T_{f}^{combo}≅14000\pm 15\%$, $T_{f}^{pd1}≅20000\pm 40\%$, and $T_{f}^{ctla4}≅13000\pm 35\%$ for the tumor cell population (yellow curves). Simulation results with the specified parameter set (σ = 0.00000022, ξ = 0.000000137, γ = 0.0000024, α = 0.0549, and β = 0.000016; all in a temporal unit of time step) show good agreement with the relative tumor-killing performance of anti-PD-1 and anti-CTLA-4 components according to cell death assessments in tumor spheroids. No unit is considered for cellular activity. Inset: Population-averaged persistence of immune cell migration in checkpoint inhibition experiments. The average values of the persistence parameter show moderate changes over time in the experiments. Error bar corresponds to standard error.

The temporal patterns of the movement activity and persistence of the cells are illustrated in Figure 4a–c. In each experiment, while the activity parameter shows considerably changing behavior, the population-averaged persistence parameter displays moderate variations over the time of observation (Figure 4a–c, insets). In the case of PD-1 blockade, the ensemble activity of the cells displays a relatively constant decrease over time from the very beginning, while blocking the CTLA-4 receptor drives the cells to a more rapid decrease in motion but after a short initial delay (Figure 4b,c). In the case of the drug combination, the migratory activity of the cells showed more intensive fluctuations but only a slight overall decrease during the time course of the experiment (Figure 4a). Additionally, as observed from time-zero values of the activities, the cells retrieved their motion strength more intensely following CTLA-4 blockade (compared with PD-1 blockade). This initial activity did not decrease significantly in combined receptor blockade, while in both cases of exclusive receptor inhibition, the activity reduction continued to approximately reach a zero value.

### 3.2. Dynamical Modeling of Lymphocyte Activity in the Presence of Checkpoint Inhibitors

The dynamics of the inferred statistical parameters was investigated to elucidate the cellular response to PD-1 and CTLA-4 checkpoint inhibitors reflected in lymphocytes’ movement behavior. Accordingly, the immobilization of immune cells in the tumor spheroids was leveraged to characterize tumor-induced T-cell suppression in the tumor microenvironment (see Section 2). The marker of the reinvigorated cells is their migration activity, measured based on the mean-square displacement (MSD) characteristics. No significant cell movement was observed in cultures without drug treatment (control samples, see Appendix A). The experimental data for the number of live tumor cells was used to evaluate the proposed model.

***Model simulation for the combination of PD-1 and CTLA-4 blockade:*** As schematically illustrated in Figure 2, in the absence of PD-1 and CTLA-4 signaling cascades, other inhibitory signals and factors in the tumor microenvironment modulate lymphocyte activity at a decreasing rate of ξ. In addition, the cytotoxic activity of T cells decreases the population of tumor cells at a rate of σ.

It was assumed that the whole population of experimentally measured spheroid tumor cells (≅ 22,000 cells±30%, Appendix A) are initially alive. Furthermore, the initial value for T-cell activity corresponds to the relevant value obtained from the autoregressive model at time 0. To infer the dynamical model parameters, we applied a Quasi-Newtonian algorithm using an unconstrained nonlinear programming solver of the MATLAB optimization toolbox (MATLAB R2016a, MathWorks). Setting the α, β, and λ parameters to zero (Equations (3)–(6), Section 2), the parameters ξ and σ were determined so that the simulation results reproduce the final tumor cell count as well as immune activity changes in this experiment (Figure 4a, blue and yellow curves). The optimal solution was found at a value of $0.55*10^{-6}\;hr^{-1}cell_{tumor}^{-1}$ for the lymphocyte inactivation rate (*ξ*) caused by factors other than the two blocked receptors. Moreover, considering the experimental value for a final tumor cell population of ≅14,000 cells ± 15%, the tumor cell death rate (*σ*) was estimated to be $0.88*10^{-6}$ $hr^{-1}Act_{imm}^{-1}$. 

***Model simulation for PD-1 blockade:*** In the absence of a CTLA-4 inhibitor, the activation of the relevant signaling pathway affects the dynamics of lymphocyte activity (Figure 2).

In this scenario, the parameters ξ and σ are set as the values inferred from the previous simulation, and similarly, the initial values are experimentally determined for this condition. In addition, tumor cell death data are used for model validation.

When training the experimental diagram, the value of $9.6*10^{-6}\;hr^{-1}cell_{tumor}^{-1}$ was estimated by the solver for the lymphocyte inactivation rate triggered by the CTLA-4 signaling cascade (γ) (Figure 4b, blue curve). As a model validation, the dynamical model in this parameter adjustment captured tumor cell death data similar to those obtained from PD-1 blockade experiments (compare the experimental value of 20,000 cells±40% with the model outcome of 19,500 cells for the final tumor cell population, Figure 4b, yellow curve).

***Model simulation for CTLA-4 blockade:*** In this case, the upregulation and consequent activation of the PD-1 pathway modify the dynamics of lymphocyte activity (Figure 2). To set the initial values of the model, we supposed that the whole population of activated lymphocytes initially belongs to the subpopulation lacking the PD-1 receptor. In this setting, the parameters α and β were learned so that the model could explain the experimental data (Figure 4c, blue curve). Because of the possibly unrealistic assumption above, we attempted to correct the initial condition of the model to obtain a more accurate estimation of the α and β parameter values. Details are given in Appendix C. 

The simulation results showed that the model is capable of reproducing the inactivation pattern of T cells for α = 0.22 $hr^{-1}$ and β = $6.33*10^{-5}\;hr^{-1}cell_{tumor}^{-1}$, which is more than six-fold greater than CTLA-4’s rate of function. The subsequent correction of the baseline situation made no significant improvement in the parameter values (Appendix C). The model was further validated by the relative concordance of the simulated cancer cell decline with the tumor-killing performance of CTLA-4 blockade (compare the final population of tumor cells in anti-PD-1 and anti-CTLA-4 treatments, Figure 4c, yellow curve). The inferred values of the parameters are represented in Table 1 (Appendix C).

***Comparing in vivo population of reinvigorated cells in PD-1 and CTLA-4 blockade:*** In addition to the investigated spheroids (average size of 70 µm [42]), we ran the model for a more realistic tumor size of about $100\;{\mathrm{mm}}^{3}$ [42], better mimicking in vivo conditions and the consequent therapeutic implications of the model.

The results demonstrate that the population of immune cells deactivated by the CTLA-4 receptor outnumbers the cells whose inactivation is associated with the PD-1 receptor by nearly ten folds (Table 1). Presuming that this model outcome is a good representation of the evolved tumor situation, this suggests that the inhibition of the CTLA-4 pathway would reactivate a larger population of immune cells than the PD-1 pathway blockade. 

Table 2 represents the average counts of cells that are set in motion following each treatment scenario. These data indicate that CTLA-4 blockade in patient-derived tumor spheroids is capable of reinvigorating a larger population of lymphocytes, which is in good agreement with the model prediction. Indeed, because of the defined time delay in the PD-1 suppressive function, a sufficiently large population of tumor cells drives immune cells to the inactivated state primarily and more efficiently by CTLA-4 receptor stimulation and signaling.

## 4. Discussion

T-cell migration can be modulated by PD-1 and CTLA-4 signaling pathways through molecular intermediates that regulate the cytoskeletal structure and membrane organization. As confirmed in our study, increasing the movement capacity to sweep the tumor microenvironment, in line with the other cell-stimulating events, is one of the results of T-cell reinvigoration following checkpoint inhibition [4,5,6,7,8,10,11].

However, controversial patterns of the response to checkpoint blockade make the identification of robust clinical biomarkers more challenging. 

In the current study, the motion pattern of tumor-associated immune cells was monitored as a functional response to PD-1 and CTLA-4 pathways to elucidate, at the cell-level temporal resolution, the kinetic constraints that potentially modulate the response to checkpoint blockade. 

Our results show that immune cells display reinforced sustained motility behavior in the presence of combined pathway inhibitors. Although such a stimulatory effect on T-cell trafficking converges with their energetic background following checkpoint inhibition, the sub-diffusive behavior of the cells, even at the beginning of the assessments, demonstrates T cells’ tendency to establish physical engagement with the tumor microenvironment (Figure 3 and Figure 4).

As shown in the activity diagrams, T-cell movement exclusively affected by PD-1 and CTLA-4 pathways depicts distinct profiles of variation, such that T cells’ mobility behavior dominated by the PD-1 pathway (anti-CTLA-4 treatment) represents a significant time lag before a continuous decline. This time delay is considered in the presented dynamical model as a time imposition of de novo PD-1 expression

This observation is supported by evidence of the profound transcriptional regulation of PD-1 and subpopulations of lymphocytes with distinct levels of PD-1 expression. Meanwhile, molecular studies have shown that CTLA-4 surface expression, independently of the transcriptional and translational rate of synthesis, lasts for several hours thanks to its cytoplasmic reserves [48,49]. 

Accordingly, the proposed dynamical model was able to explain the distinct immunostimulatory pattern of PD-1 and CTLA-4 blockade based on mechanisms involved in the regulation of their surface expression. Importantly, the model could explain how, despite the functionally more efficient signaling cascade initiated by the PD-1 signal (compare the parameter values in Table 1), the presence of cytoplasmic vesicles storing CTLA-4 receptors enhances its suppressive function and eventually leads to the dominance of its effects at the level of lymphocyte populations.

Our analyses suggest that the relative population of immune cells downregulated by these two pathways depends on the initial population size of the interacting tumor cells. In tumors with relatively large baseline sizes, the outstanding time delay in PD-1 expression potentially leads to exhaustive immune inactivation as the result of CTLA-4 function, which shapes an inherent limitation for anti-PD-1 treatment outcomes. Consistent with this notion, our study emphasizes the importance of baseline tumor size as a marker of the response to anti-PD-1 and anti-PD-L-1 treatment. While the antigen burden (i.e., tumor mutation burden) is already considered a possible explanation for the tumor-size effect, our study suggests that even in spite of the pretreatment adaptation of tumor–immune populations, the marker of tumor size remains a limiting factor for the patient response to anti-PD-1 and anti-PD-L-1 treatments [50,51,52].

It should be noted that this relatively sharp immune stimulation may lead to the activation of other immunosuppressive pathways and enhance the restoration of immunoregulatory cells, which explains the possibility of poor therapeutic outcomes in response to CTLA-4 blockade [53,54,55]. Indeed, our analyses further emphasize the importance of pretreatment immune profiling for anti-CTLA-4-based treatments and highlight the significance of identifying predictive biomarkers as a strict irrevocable goal for improving the response to CTLA-4 blockade.

On the other hand, considering the importance of the initial response and the priming of anti-tumor immunity for immunotherapy outcomes, the profound immune excitation predicted here provides further rationales for combined and alternative strategies for treatment [56]. Treating patients with immunostimulatory agents with acceptable immune-related toxicity following the initial activation of the immune system by CTLA-4 blockade may improve clinical outcomes. Moreover, in patients without baseline population-related biomarkers for anti-PD-1 treatment [35,54], this strong immune restoration may establish conditions for an improved objective response. Previous studies have also confirmed the significance of immunotherapy scheduling in the kinetic pattern of immune stimulation as a result of nonlinear effects in the context of glioma–immune interaction [31,32,57,58,59].

The presented quantitative insight into the distinct PD-1 and CTLA-4 functions can be useful for the development of more relevant models for planning effective treatment schemes. More generally, this study highlights the need for a more detailed understanding of the kinetics of the response to checkpoint inhibitors toward developing therapeutic strategies based on the multimodal stimulation of the immune response.

## Figures and Tables

**Table 1:** Tumor–immune interaction parameters.

Parameter	Definition	*OptimalValue* *	*Unit* **
σ	Tumor cell death rate	$8.8*10^{-7}$	$hr^{-1}Act_{imm}^{-1}$
γ	CTLA-4-induced immune cell inactivation rate	$9.6*10^{-6}$	$hr^{-1}cell_{tumor}^{-1}$
β	PD-1-induced immune cell inactivation rate	$6.33*10^{-5}$	$hr^{-1}cell_{tumor}^{-1}$
ξ	Non-checkpoint-induced immune cell inactivation rate	$5.5*10^{-7}$	$hr^{-1}cell_{tumor}^{-1}$
α	PD-1 expression rate	0.22	$hr^{-1}$

* Optimal values estimated by fitting the model simulation to corresponding experimental results. ** Accounting for time intervals corresponding to 15 min and unit-free measure of cell activity.

**Table 2:** Reinvigorated cell counts in checkpoint blockade experiments.

Type of Treatment	Spheroids’ Active Cell Number (Average)
Anti-CTLA-4	65.6 ± 9.38
Anti-PD-1	19.3 ± 1.64
Combo	61.6 ± 7.75

**Figure A1:**
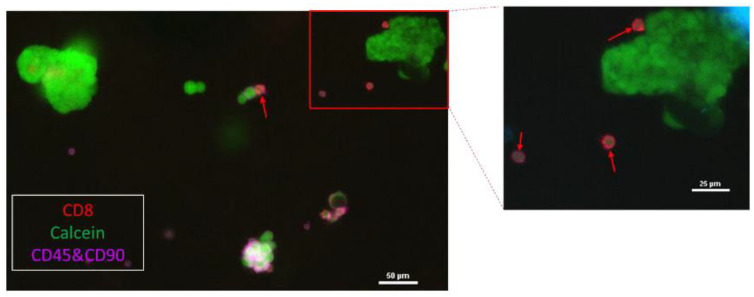
**Immune profiling and viability evaluation of patient-derived thyroid tumor spheroids.** A representative image of immunofluorescence staining that identifies the population of autologous CD45+ and CD8+ immune cells in organotypic tumor spheroids derived from patient samples.

## Data Availability

The data generated and analyzed during the current study are available at https://github.com/safaeifard/ (accessed on 26 March 2022).

## Appendix A

### Experimental Background

The derivation, treatment, and characterization of tumor spheroids are described in detail in the article previously published by Jenkins et al. (*Cancer Discovery* 8.2 (2018)). Briefly, during the experimental procedure, patient-derived tumor spheroids in combination with collagen were loaded into the central channel of a 3D microfluidic culture device (Figure 1, main text). The cell culture was treated with monoclonal antibodies or their combinations. Pembrolizumab and Ipilimumab antibodies were used as PD-1 and CTLA-4 pathway inhibitors, respectively. The injected dose (clinically used 1:100 dilutions of stock concentrations) resulted in peak plasma concentrations measured following the administration of 10 mg/kg of each drug (FDA CDER application). In these experiments, the immune profiles of tumor spheroids were characterized by different immune markers detected by the flow cytometry technique.

The presence of different cytokines and their concentrations in the cell culture microenvironment were evaluated using a bead-based immunoassay approach. These outcomes of assays show the success of the designed cell culture in recapitulating the tumor microenvironment, considering its autologous cell population and chemical signals.

In addition, live/dead cell quantification was performed using dual-labeling deconvolution fluorescence microscopy. The presence of a lateral channel along the central area of the culture medium entails the possibility of controlled drug injection. Time-lapse imaging of cell mobility was carried out through the transparent cover of the chamber. All of the experiments were performed at Dana–Farber Cancer Institute. Below, the relevant experimental protocols and results are discussed.

***Tumor samples:*** The immune cells were investigated in organotypic cultures of tumor spheroids (including lymphoid and myeloid cells) derived from patient samples of thyroid and melanoma cancers. Depending on the patient’s response to ICB components, the spheroids were cultured in four types of mediums containing: (1) PD-1 and (2) CTLA-4 inhibitors and (3) their combination alongside (4) control cultures.

***Live/Dead imaging of tumor spheroids****:* With the goal of tumor and non-tumor cell death quantification, live/dead imaging was performed using dual-labeling deconvolution fluorescence microscopy (Figure A1). The results are shown in Table A1. These data were used to calculate the initial and final populations of tumor cells (TDR:TumorDeathRatio):

$$\begin{array}{cc} TumorPopulation_{t=0}= & AverageSpheroidsPopultion*\\ & AverageSpheroidsTumorRatio*\left(1-TDR_{control}\right), \end{array}$$

$$\begin{array}{cc} TumorPopulation_{final}= & TumorPopulation_{t=0}-\frac{TDR_{treatment}-TDR_{control}}{1-TDR_{control}}*\\ & TumorPopulation_{t=0}, \end{array}$$

which gives the initial and final values of the tumor cell population:

$$T_{t_{0}}≅22000\pm 30\%,$$

$$T_{f}^{combo}≅14000\pm 15\%,$$

$$T_{f}^{pd1}≅20000\pm 40\%,$$

$$T_{f}^{ctla4}≅13000\pm 35\%,$$

**Table A1:** Spheroid live/dead and tumor–immune profiling.

Experiment	Spheroid Population	Tumor Cell Ratio *	Tumor Death Ratio **
Control	175000±25%	0.22	0.090
PD-1	175000±30%	0.16	0.196
CTLA4	130000±25%	0.16	0.483
Combo	180000±30%	0.13	0.433

* The ratio of tumor cells to the total population of spheroid cells (tumor + immune cells) for each drug treatment. ** The ratio of tumor cells to the total tumor cell population of spheroids (dead + live cells).

***Time-lapse imaging of tumor culture:*** Time-lapse images of bright-field microscopy were obtained during the experiments. The images were captured every 15 min using a Nikon Ti inverted microscope with a 10× NA 0.3 objective and cooled CCD camera (Orca R2, Hamamatsu) in a humidified, temperature-controlled chamber. Illumination was with a CoolLED pe-100 white-light LED. Imaging of various fields of view over time was controlled by NIS-Elements software of a Prior motorized stage along with the LED and camera. Image capturing was carried out for more than 30 h (Figure 1).

***Activated cell count in each treatment:*** The number of lymphocytes that were set in motion was estimated at the beginning of PD-1 and CTLA-4 pathway blockade. For this aim, we used time-lapse microscopic images of the first few frames to count moving cells in microfluidic cell cultures treated with each blocking agent.

## Appendix B

### Evaluation of Parameter Estimation for AR-1 Model of T-Cell Migration

A sequential Bayesian inference method corresponding to the autoregressive model of the first order was applied to the cell trajectories to deduce the joint probability distribution of the model parameters for each time step (Figure 1). We used the average values of the inferred distributions to trace statistical parameter changes over the single-cell migration period.

To evaluate the method and the parameter estimation algorithm for our migration assay, single-cell time-varying parameters were used in an inverse manner to see if the model can reproduce trajectories that statistically match the migration data.

The results of parameter estimation for simulated trajectories indicated that the Bayesian method and the applied algorithm are able to conserve the input temporal changes in the model parameters in simulated trajectories (Figure A2).

## Appendix C

### Appendix C.1. Parameter Inference for the Random Walk Model of Lymphocyte Migration

We used a Bayesian inference approach based on discretized probability distributions presented by Metzner, C. et al. [43] for the joint inference of the activity (a) and persistence (p) parameters in the AR-1 model of migration. Considering the two-dimensional vector of cellular velocities, the likelihood function of this process can be considered as follows:

$$P\left(u_{t+1}|p_{t+1},a_{t+1};u_{t}\right)=\frac{1}{2\pi a_{t+1}^{2}}\exp\left(-\frac{{\left(u_{t+1}-p_{t+1}u_{t}\right)}^{2}}{2a_{t+1}^{2}}\right)$$

The likelihood function is sequentially evaluated for the measured position of the cells in each time step $u_{t+1}$, given the previous cell state $u_{t}$ and the optimal values for the latent parameters.

Starting from a flat distribution $P_{0}$, the numerical values of the likelihood function are sequentially multiplied by the corresponding discrete points of the $\left(p_{t},a_{t}\right)$-grid to update the prior distribution. The time series of the averaged values of the posterior distributions are considered temporal variations in the activity and persistence of the cells to use in the subsequent analysis. The minimal probability of the posterior distribution and the grid size were set to $P_{min}$ = 10−10 and 200, respectively. See [43] for more details.

### Appendix C.2. Estimation of Parameters for the Dynamical Model of Checkpoint-Induced Lymphocyte Inactivation

The integrated dynamical model presented in Equations (3)–(6) has five unknown parameters. The list of parameters and associated dimensions are given in Table 1. There are four time series of experimental observations to which the modeling results should be fit: the lymphocyte activity changes in each checkpoint-blockade experiment in addition to the initial and final tumor cell population in anti-PD-1 + anti-CTLA-4 treatment.

In order to find an optimal parameter set that reproduces the experimentally extracted data points, we used a nonlinear unconstrained optimization workflow to minimize the following cost function O(π):

$$\min\sum_{t=0}^{k}{\left(Y\left(t\right)-Y_{\pi }\left(t\right)\right)}^{2}$$

where Y(t) indicates the observed data point in time step t, and $Y_{\pi }\left(t\right)$ is the corresponding theoretical value obtained from the model given the parameter set π. The number of data points observed in each experiment is denoted by k.

Starting from an initial guess, the solution space of the parameters is explored for the optimal values, which are given iteratively to an ordinary differential equation solver for the evaluation of the model. We used the Quasi-Newtonian algorithm to minimize the above cost function. All of the analyses were performed using MATLAB optimization methods and toolbox (MATLAB R2016a, MathWorks).

### Appendix C.3. Improving the Initial Values of Lymphocyte Subpopulations (Correction of α and β Parameters)

Considering that the initial values of activity for the three subpopulations of lymphocytes (PD-1-deficient lymphocytes, those partially expressing PD-1, and those fully expressing this receptor) might have been unrealistic, the integrated model was run for a better estimation of the α and β parameters. In order to do so, using the already estimated α and β parameters, the model was simulated, assuming again that the total activity initially belongs to the unexpressed subpopulation.

The model-derived distribution of CTLA-4-deactivated cells with different PD-1 expression states was compared. It was anticipated that the relative populations would give a better estimation for the initial values of the simulation, which would result in a more accurate estimation of the α and β parameters under the condition of CTLA-4 inhibition.

However, the dominant proportion of the lymphocyte population gathered in the stock of the subpopulation lacking the PD-1 receptor. Moreover, there was no improvement in the estimation of the α and β parameters when we used the obtained distribution of lymphocytes as initial values. Therefore, the presumption (model hypothesis) that initially assigned the total activity to the population lacking the PD-1 receptor is an admissible assumption.
